# Wheatgrass-and-Aronia-Mixed Extract Suppresses Immunoglobulin E-Mediated Allergic Reactions In Vitro and In Vivo

**DOI:** 10.3390/ijms241511979

**Published:** 2023-07-26

**Authors:** Ji-Hyun Lee, Ji-Ye Lim, Yong-Deok Jeon, Dae-Ho Yun, Young-Mi Lee, Dae-Ki Kim

**Affiliations:** 1 Department of Immunology, Jeonbuk National University Medical School, Jeonju-si 54907, Republic of Korea; jihyunsh1211@naver.com (J.-H.L.); 84juce@naver.com (J.-Y.L.); 2 Department of Korean Pharmacy, Woosuk University, Wanju-Gun, Samnye-eup 55338, Republic of Korea; ydjeon1211jh@woosuk.ac.kr; 3 Department of Health Administration, Kwangju Women’s University, Kwangju 62396, Republic of Korea; ydh016@hanmail.net; 4 Department of Oriental Pharmacy, College of Pharmacy, Wonkwang-Oriental Medicines Research Institute, Wonkwang University, Iksan 54538, Republic of Korea; ymlee@wku.ac.kr

**Keywords:** *Triticum aestivum* L., *Aronia melanocarpa*, allergic inflammation, immunoglobulin E, oxidative stress, anaphylaxis

## Abstract

Mast cells are an important component of immune responses. Immunoglobulin (Ig) E-sensitized mast cells release substances within minutes of allergen exposure, triggering allergic responses. Until now, numerous pharmacological effects of wheatgrass and aronia have been verified, but the effects of wheatgrass and aronia (TAAR)-mixed extract on allergic reactions have not been identified. Therefore, the aim of this study was to demonstrate the anti-allergic effect of TAAR extract on mast cell activation and cutaneous anaphylaxis. In this study, we investigated the anti-allergic effects and related mechanisms of TAAR extract in IgE-activated mast cells in vitro. We also assessed the ameliorating effect of TAAR extract on IgE-mediated passive cutaneous anaphylaxis mice in vivo. The TAAR extract significantly reduced the expression of β-hexosaminidase, histamine, and pro-inflammatory cytokines, which are mediators related to mast cell degranulation, via the regulation of various signaling pathways. The TAAR extract also regulated oxidative-stress-related factors through the Nrf2 signaling pathway. Additionally, treatment of TAAR extract to the passive cutaneous anaphylaxis mouse model improved ear thickness and local ear pigmentation. Taken together, our results suggest that TAAR extract is a potential candidate natural product to treat overall IgE-mediated allergic inflammation and oxidative-stress-related diseases by suppressing mast cell activity.

## 1. Introduction

The prevalence of type I allergic diseases such as allergic rhinitis, anaphylaxis, drug or food allergies, asthma, and atopic dermatitis is steadily increasing, particularly in developed countries. Allergic diseases can reduce the quality of life of many patients and account for approximately 25% of the medical expenses in developed countries; thus, they significantly contribute to the continuous rise cost of healthcare and are an emerging global concern [1].

When exposed to allergens, hypersensitivity of the body’s immune system is induced, and immunoglobulin E (IgE) is produced, which adheres to the high-affinity IgE receptor (FcεRI) on mast cells or basophils in target tissues [2]. When the IgE-FcεRI complex on the cell surface is re-exposed to an allergen during an IgE-mediated allergic reaction, cell degranulation is induced through intracellular signaling and the immediate secretion of β-hexosaminidase, histamine, and various inflammatory mediators, including cytokines [3]. These mediators can cause acute allergic reactions with potentially life-threatening consequences. Accordingly, these mediators are major targets for effective allergic disease treatment, and medications such as antihistamines, glucocorticoids, steroids, and mast cell stabilizers have been developed [4]. However, almost all drugs commonly used for allergy treatment have unexpected side effects, such as drowsiness, sedation, and weight gain; therefore, long-term use is unsustainable, and treatment is limited because they only temporarily relieve the symptoms of allergic inflammation [5]. Therefore, the development of allergic therapeutic agents using edible plant materials with low side effects and high efficacies is attracting attention, and safe allergy treatments using these agents are required. Based on previous research, we selected wheatgrass and aronia as candidate plant materials for potential allergy treatments and decided to mix them in anticipation of their better effects.

Wheat (*Triticum aestivum* L., TA) is a major global food crop and an essential source of human nutrition because it is rich in proteins, starch, minerals, dietary fiber, and phenolic compounds [6]. Wheat sprouts contain high concentrations of plant nutrients, such as various physiologically active substances that protect them from external attacks during germination [7]. Wheatgrass has recently received considerable attention as a healthy food supplement. Many studies have reported that wheatgrass has anti-inflammatory [8] and anti-oxidant properties [9] and is effective in relieving several diseases such as diabetes [10], liver damage [11,12], obesity [13], atopic dermatitis [14], allergies [15], and ulcerative colitis [16].

*Aronia melanocarpa* (AR), also known as black wild chokeberry, is native to North America. Aronia became famous in Russia in the 20th century and is consumed mainly as fruit, jam, and wine, although it has also been used as an ingredient in traditional medicine [17]. Aronia contains many nutrients such as proteins, minerals, lipids, and high levels of polyphenols [18]. Previous studies have reported that aronia has anti-inflammatory [19,20], antioxidant, and antiviral effects [21,22].

Despite the various benefits and potential therapeutic effects of wheatgrass and aronia, their effects on allergic reactions have yet to be confirmed. Wheatgrass-and-aronia-mixed extracts were reported to have a positive effect on alleviating symptoms of atopic dermatitis [23], and this study aimed to determine if the physiological characteristics of the wheatgrass-and-aronia-mixed extract could control allergic inflammatory responses and to identify the related mechanism. The experimental results of this study serve as the basis for selecting the optimal mixing wheatgrass and aronia ratio.

## 2. Results

### 2.1. The Mixing Ratio Affects the Anti-Inflammatory Effect of Wheatgrass and Aronia Mixed Extracts

**Figure 1:**
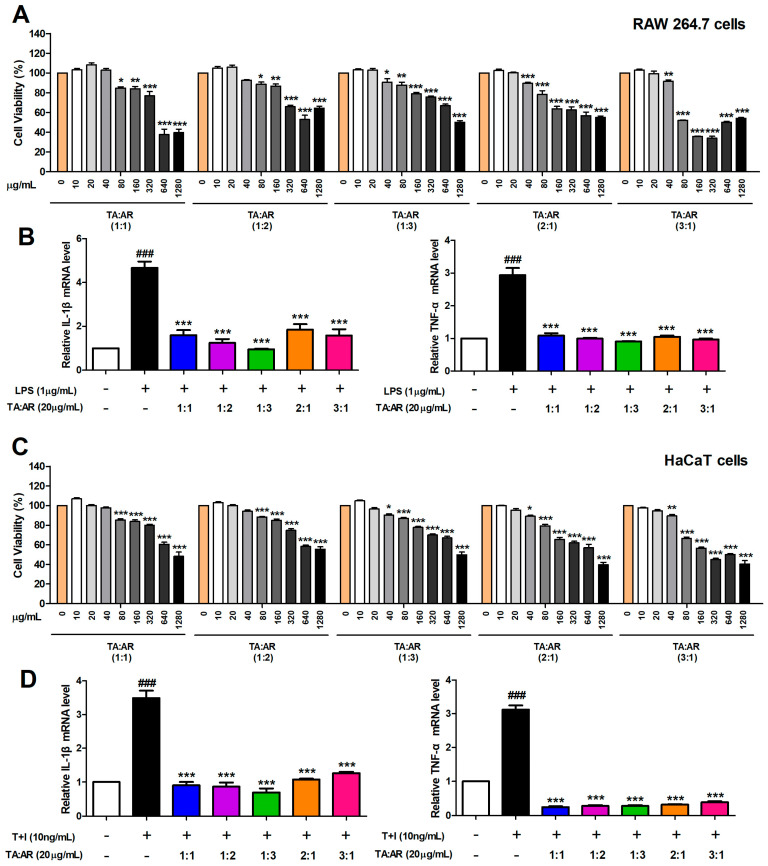
Anti-inflammatory effects of mixed wheatgrass-and-aronia-mixed extracts depend on the ratios. (**A**) Cell viability of RAW 264.7 cells in response to treatment with wheatgrass-and-aronia-mixed extracts at different mixing ratios. (**B**) The mRNA expression levels of pro-inflammatory cytokines in response to treatment with mixing ratios of wheatgrass-and-aronia-mixed extracts in LPS-induced RAW 264.7 cells. (**C**) Cell viability of HaCaT cells in response to treatment with wheatgrass-and-aronia-mixed extracts at different mixing ratios. (**D**) The mRNA expression levels of pro-inflammatory cytokines in response to treatment with wheatgrass-and-aronia-mixed extracts at different mixing ratios in TNF-α/IFN-γ-induced HaCaT cells. Values are represented as the mean ± SEM and analyzed by Tukey’s post hoc test from three independent experiments. ^###^ *p* < 0.001 vs. no treatment control group; * *p* < 0.05, ** *p* < 0.01, and *** *p* < 0.001 vs. LPS- or TNF-α/IFN-γ treatment only group. TA, wheatgrass; AR, aronia.

To evaluate how the mixing ratio of wheatgrass and aronia affects cell viability, the RAW 264.7 mouse macrophage cell line and human keratin HaCaT cell line were treated with the wheatgrass-and-aronia-mixed extract at various mixing ratios (1:1, 1:2, 1:3, 2:1, and 3:1) and concentrations (0, 10, 20, 40, 80, 160, 320, 640, and 1280 μg/mL) for 24 h before cell viability was measured using the MTT assay. We found no cytotoxicity in both cell lines in the concentration range of 10–40 μg/mL for 1:1 and 1:2 ratios of wheatgrass-and-aronia-mixed extracts. In addition, there was no cytotoxicity in the concentration range of 10–20 μg/mL for the 1:3, 2:1, and 3:1 combination ratios of wheatgrass-and-aronia-mixed extracts (Figure 1A,C). Based on these results, 20 μg/mL of the mixed wheatgrass and aronia extract was used in subsequent experiments. Real-time PCR was performed to analyze whether the wheatgrass-and-aronia-mixed extracts suppressed the mRNA expression of pro-inflammatory cytokines. The expression levels of pro-inflammatory cytokines (TNF-α and IL-1β) were significantly decreased in both cell lines after treatment with various mixing ratios of wheatgrass-and-aronia-mixed extract (Figure 1B,D).

### 2.2. The Mixing Ratio of Wheatgrass and Aronia Extract Is Associated with an Antioxidant Effect

**Figure 2:**
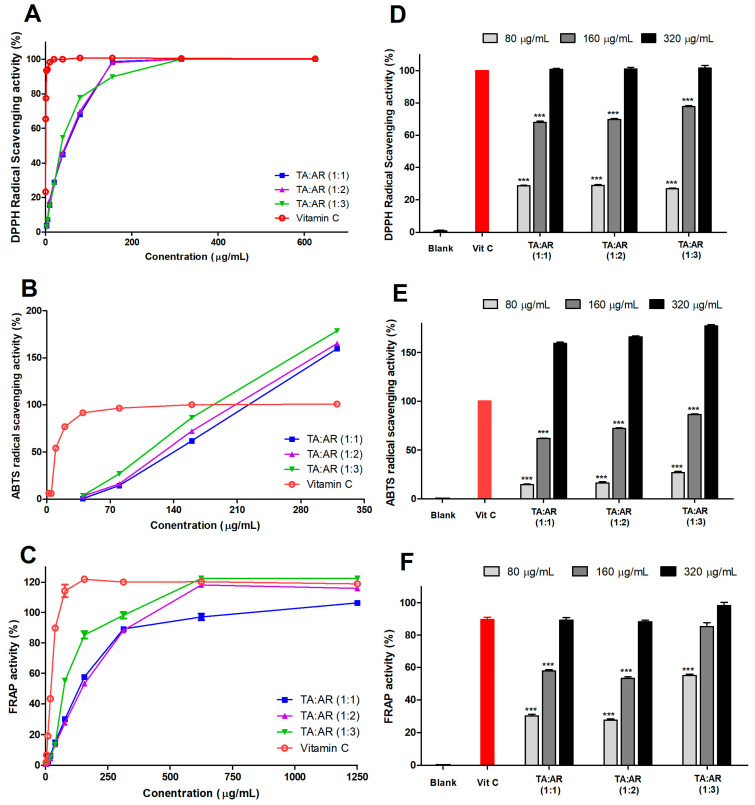
Antioxidant activity of wheatgrass-and-aronia-mixed extracts is dependent on the mixing ratio and concentration. (**A**,**D**) DPPH and (**B**,**E**) ABTS radical scavenging activity in samples treated with different wheatgrass and aronia mixing ratios and concentrations. (**C**,**F**) FRAP activity in samples treated with different wheatgrass and aronia mixing ratios and concentrations. Vitamin C was used as a positive control. Each value is represented as the mean ± SEM and analyzed by Tukey’s post hoc test from three independent experiments. *** *p* < 0.001 vs. vitamin C. TA, wheatgrass; AR, aronia.

Assays measuring DPPH, ABTS, and FRAP activity were used to confirm the antioxidant effects of the wheatgrass-and-aronia-mixed extracts according to changes in aronia content. The antioxidant activities of various concentrations of wheatgrass-and-aronia-mixed extracts at mixing ratios of 1:1, 1:2, and 1:3 were compared using vitamin C (4 μg/mL) as a positive control. The antioxidant activities of the wheatgrass and aronia extracts at all mixing ratios increased in a concentration-dependent manner. In addition, as the aronia content increased in the wheatgrass-and-aronia-mixed extracts at the same concentration, the antioxidant effect also increased (Figure 2).

### 2.3. Quantification of Active Components of the TAAR Extract by HPLC

**Figure 3:**
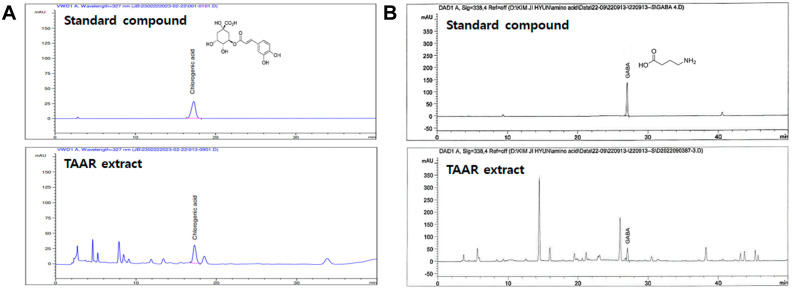
HPLC analysis of the TAAR extract. HPLC chromatograms of the (**A**) chlorogenic acid and (**B**) GABA standard compounds and TAAR extract samples.

Based on the above results, the research team concluded that a 1:3 mixing ratio of wheatgrass to aronia had the best anti-inflammatory and antioxidant effects. Therefore, a mixed extract of wheatgrass and aronia in a 1:3 ratio (TAAR extract) was selected and used in subsequent experiments, and HPLC was conducted to verify the main components of the TAAR extract (Figure 3). This analysis confirmed that the main components of the TAAR extract were chlorogenic acid and GABA, which were identified by comparison with the peaks of the internal standard components. The concentrations of chlorogenic acid and GABA in the TAAR extract were 0.2193 and 0.2155 mg/g, respectively.

### 2.4. TAAR Extract Reduces Mast Cell Degranulation and Mast Cell-Mediated Pro-Inflammatory Cytokine Secretion in Anti-DNP IgE Plus DNP-BSA-Induced RBL-2H3 Cells

**Figure 4:**
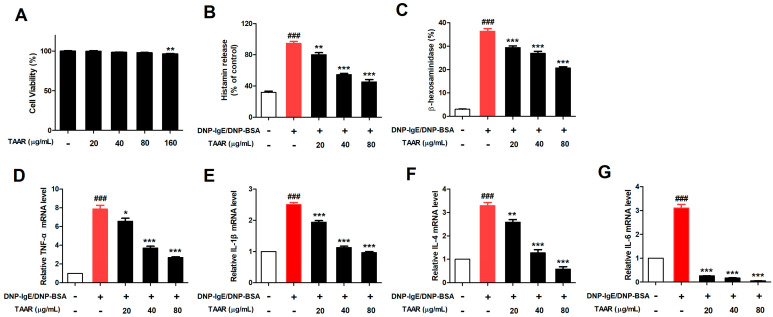
Effects of TAAR extract treatment on cell viability, degranulation, and inflammatory cytokine expression in mast cells. (**A**) Cell viability of RBL-2H3 cells after TAAR extract treatment was measured using MTT assay. The (**B**) histamine and (**C**) β-hexosaminidase levels of anti-DNP IgE/BSA-induced RBL-2H3 cells after TAAR extract treatment. (**D**–**G**) The mRNA levels of pro-inflammatory cytokine genes TNF-α, IL-1β, IL-4, and IL-6 in anti-DNP IgE/BSA-induced RBL-2H3 cells were measured using real-time PCR. Each value is presented as the mean ± SEM and analyzed with Tukey’s post hoc test from three independent experiments. ^###^ *p* < 0.001 vs. no treatment control group; * *p* < 0.05, ** *p* < 0.01, and *** *p* < 0.001 vs. anti-DNP IgE/BSA treatment only group.

To confirm the cytotoxicity of the TAAR extract on mast cells, RBL-2H3 cells were treated with 0, 20, 40, and 80 µg/mL of the TAAR extract for 24 h and cell viability was evaluated with an MTT assay. No significant cytotoxicity was observed in the tested concentration range of the TAAR extract in the RBL-2H3 cells (Figure 4A); therefore, subsequent experiments were performed using the TAAR extract at concentrations ranging from 0 to 80 µg/mL. Increased histamine and β-hexosaminidase secretion are representative features of mast cell activation [3]. We used RBL-2H3 cells sensitized with anti-DNP IgE and DNP-BSA to investigate the effect of TAAR extract on IgE-mediated allergic responses in mast cells. Anti-DNP IgE and DNP-BSA sensitization increased histamine and β-hexosaminidase secretion in RBL-2H3 cells. However, treatment with TAAR extract significantly decreased the secretion of histamine and β-hexosaminidase in a concentration-dependent manner (Figure 4B,C). Additionally, we investigated the effect of the TAAR extract on pro-inflammatory cytokine production in activated mast cells using anti-DNP IgE and DNP-BSA. The TAAR extract significantly reduced the production of TNF-α, IL-1β, IL-4, and IL-6, suggesting that the TAAR extract had anti-degranulation activity in activated mast cells and alleviated the allergic inflammatory response by suppressing the production of pro-inflammatory cytokines (Figure 4D–G).

### 2.5. TAAR Extract Inhibits Mitogen-Activated Protein Kinase (MAPK) Signaling Pathway Activation in Anti-DNP IgE Plus DNP-BSA-Induced RBL-2H3 Cells

**Figure 5:**
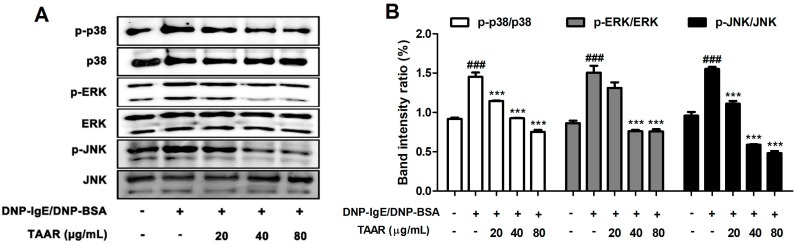
Effects of the TAAR extract on the activation of MAPK signaling pathways in mast cells. (**A**) The protein expression levels of phosphorylated and total forms of MAPK signaling pathway proteins in anti-DNP IgE/BSA-treated RBL-2H3 cells. (**B**) Bar graphs displaying the relative intensity ratios of Western blot bands for p-p38/p38, p-ERK/ERK, and p-JNK/JNK. Each value is presented as the mean ± SEM and analyzed by Tukey’s post hoc test from three independent experiments. ^###^ *p* < 0.001 vs. no treatment control group; *** *p* < 0.001 vs. anti-DNP IgE/BSA treatment only group.

As the TAAR extract significantly suppressed pro-inflammatory cytokines, we further investigated the effect of the TAAR extract on the MAPK (p38, ERK, and JNK) signaling pathway, which is part of an inflammation-related mechanism [24]. As shown in Figure 5A,B, all proteins involved in the MAPK signaling pathway were phosphorylated in mast cells activated by anti-DNP IgE and DNP-BSA. However, treatment with the TAAR extract significantly decreased the phosphorylation of p38, ERK, and JNK in the anti-DNP IgE and DNP-BSA-induced RBL-2H3 cells. These results suggested that the TAAR extract could effectively control the allergic response by alleviating the secretion of pro-inflammatory cytokines via the inhibition of the MAPK signaling pathway.

### 2.6. TAAR Extract Attenuates Nuclear Factor-κB (NF-κB) Nuclear Translocation and Related Inflammatory Mediators in Anti-DNP IgE Plus DNP-BSA-Induced RBL-2H3 Cells

**Figure 6:**
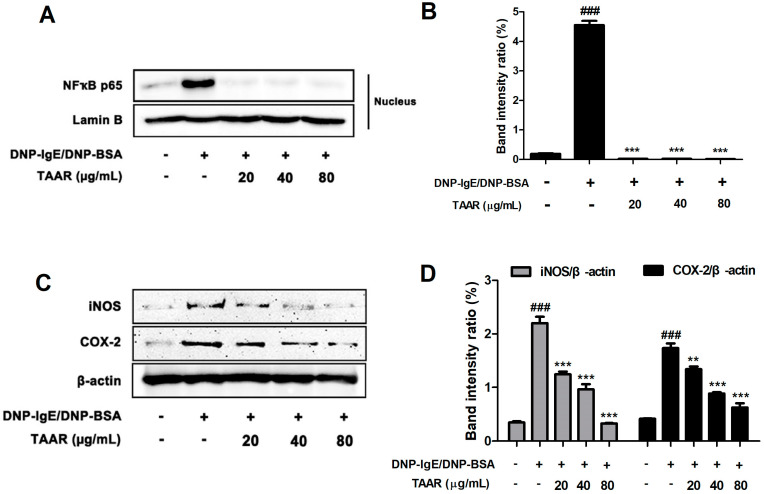
Effects of the TAAR extract on NF-κB nuclear translocation and inflammatory mediators in mast cells. (**A**) The nucleus NF-κB protein expression levels in anti-DNP IgE/BSA-treated RBL-2H3 cells. (**B**) Bar graph displaying relative intensity ratios of Western blot bands for NF-κB. (**C**) The protein expression levels of iNOS and COX-2 in anti-DNP IgE/BSA-treated RBL-2H3 cells. (**D**) Bar graphs displaying the relative intensity ratios of Western blot bands for iNOS/β-actin and COX-2/β-actin. Each value is presented as the mean ± SEM and analyzed by Tukey’s post hoc test from three independent experiments. ^###^ *p* < 0.001 vs. no treatment control group; ** *p* < 0.01 and *** *p* < 0.001 vs. anti-DNP IgE/BSA treatment only group.

Oxidative stress and inflammation play an important role in the development of diseases like allergies. Therefore, we investigated the effect of TAAR extract on the regulation of NF-κB nuclear translocation, a mechanism related to oxidative stress and inflammation. As a result, the expression of NF-κB in the nucleus of RBL-2H3 cells stimulated with anti-DNP IgE and DNP-BSA was up-regulated, but the expression of NF-κB in the nucleus was significantly down-regulated by TAAR extract treatment (Figure 6A,B). In addition, TAAR extract notably reduced the expression of inflammatory mediators such as iNOS and COX-2, which were increased by anti-DNP IgE and DNP-BSA in RBL-2H3 cells (Figure 6C,D). These results indicate that TAAR extract alleviates the secretion of inflammatory mediators by blocking the nuclear translocation of NF-κB.

### 2.7. TAAR Extract Regulates the Activation of Nrf2/HO-1/NQO1 Signaling Pathway in Anti-DNP IgE Plus DNP-BSA-Induced RBL-2H3 Cells

**Figure 7:**
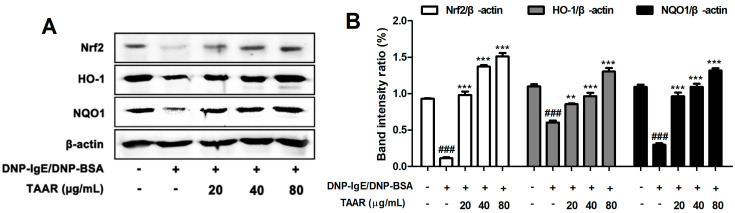
Effects of the TAAR extract on Nrf2 activation signaling pathway in mast cells. (**A**) The protein expression levels of Nrf2, HO-1, and NQO1 in anti-DNP IgE/BSA-treated RBL-2H3 cells. (**B**) Bar graphs displaying the relative intensity ratios of Western blot bands for Nrf2/β-actin, HO-1/β-actin, and NQO1/β-actin. Each value is presented as the mean ± SEM and analyzed by Tukey’s post hoc test from three independent experiments. ^###^ *p* < 0.001 vs. no treatment control group; ** *p* < 0.01 and *** *p* < 0.001 vs. anti-DNP IgE/BSA treatment only group.

Oxidative stress results in the nuclear translocation of Nrf2, which is activated to promote the expression of antioxidant and detoxification enzyme genes such as HO-1 and NQO1. Up-regulation of these genes plays a role in protecting cells against oxidative stress. Therefore, we confirmed the expression of oxidative stress-related factors such as Nrf2, HO-1, and NQO1 in order to examine whether TAAR extract exhibits antioxidant effects in mast cells under oxidative stress. As a result, as shown in Figure 7, it was confirmed that the expression of Nrf2, HO-1, and NQO1, which were reduced by stimulation with anti-DNP IgE and DNP-BSA, was increased in a concentration-dependent manner by treatment with TAAR extract. These results indicate that TAAR extract improves oxidative stress in mast cells.

### 2.8. TAAR Extract Decreases the Activation of FcεRI Signaling Cascade in Anti-DNP IgE Plus DNP-BSA-Induced RBL-2H3 Cells

**Figure 8:**
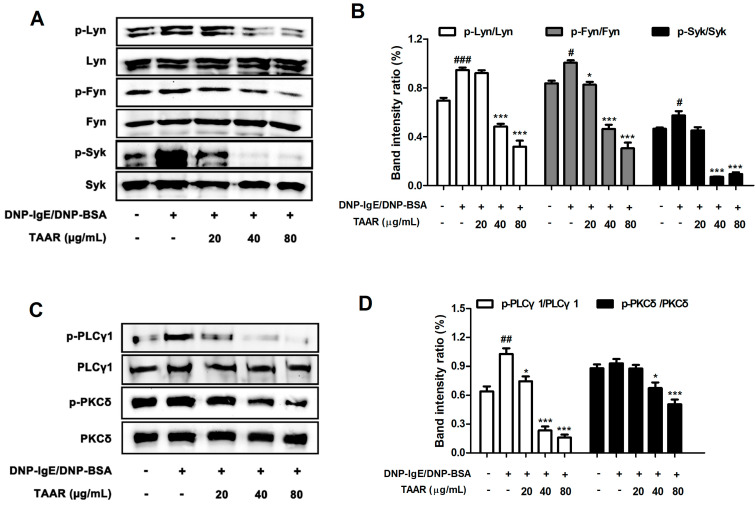
Effects of TAAR extract treatment on FcεRI signaling cascade protein expression in mast cells. (**A**) The protein expression levels of p-Lyn, Lyn, p-Fyn, Fyn, p-Syk, and Syk in anti-DNP IgE/BSA-treated RBL-2H3 cells. (**B**) Bar graphs displaying the relative intensity ratio of Western blot bands for p-Lyn/Lyn, p-Fyn/Fyn, and p-Syk/Syk. (**C**) The protein expression of p-PLCγ1, PLCγ1, p-PKCδ, and PKCδ in anti-DNP IgE/BSA-treated RBL-2H3 cells (**D**) Bar graphs displaying the relative intensity ratio of Western blot bands for p-PLCγ1/PLCγ1, and p-PKCδ/PKCδ. Each value is presented as the mean ± SEM and analyzed by Tukey’s post hoc test from three independent experiments. ^#^ *p* < 0.05, ^##^ *p* < 0.01, and ^###^ *p* < 0.001 vs. no treatment control group; * *p* < 0.05 and *** *p* < 0.001 vs. anti-DNP IgE/BSA treatment only group.

Western blotting was performed to evaluate the regulatory effect of the TAAR extract on the activation of the signaling cascade in the early stage of the IgE-FcεRI signaling pathway in mast cells. Phosphorylation of Lyn, Fyn, and Syk occurs immediately upon FcεRI stimulation in mast cells and plays a crucial role in the initiation of mast cell activation and degranulation [25]. The phosphorylation levels of Lyn, Syk, and Fyn were significantly up-regulated by anti-DNP IgE and DNP-BSA stimulation but suppressed by treatment with the TAAR extract (Figure 8A,B). In particular, concentrations of 40 µg/mL and 80 µg/mL TAAR extract showed significantly inhibitory effects. We next investigated the inhibitory effect of the TAAR extract on the phosphorylation of PLCγ1 and PKCδ, which are associated with a related mechanism known to stimulate the secretion of histamine, β-hexosaminidase, and inflammatory cytokines [24]. We found that the phosphorylation of PLCγ1 was significantly increased by stimulation with anti-DNP IgE and DNP-BSA, and the phosphorylation of PKCδ was increased but not significant. TAAR extract down-regulated the elevated phosphorylation of PLCγ1 and PKCδ in anti-DNP IgE- and DNP-BSA-stimulated mast cells (Figure 8C,D). Taken together, these results suggest that TAAR extract can effectively prevent IgE-mediated allergic reactions through the inhibition of FcεRI-mediated mast cell activation by down-regulating the phosphorylation of Lyn, Syk, Fyn, PLCγ1, and PKCδ.

### 2.9. TAAR Extract Weakens the IgE-Mediated Cutaneous Anaphylaxis Reaction in Mice

**Figure 9:**
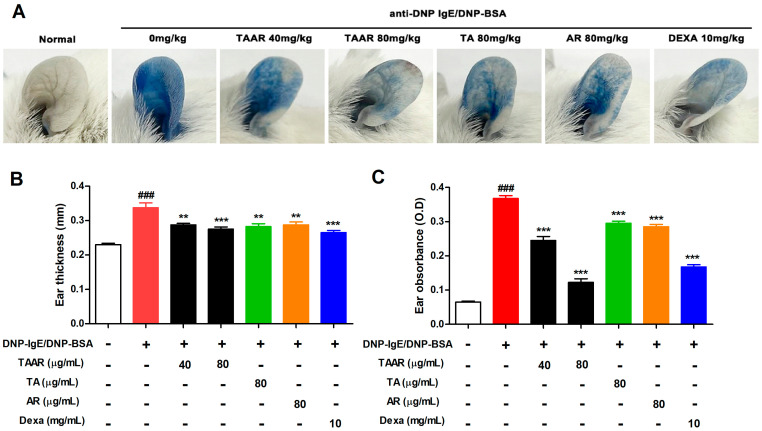
Effects of TAAR extract on IgE-mediated passive cutaneous anaphylaxis a mouse model. (**A**) Representative pictures of the ear skin of mice in each treatment group (n = 6 mice per group, 30 min time). (**B**) Dye was extracted from the ears of mice from each treatment group and quantified using a spectrophotometer. (**C**) Ear thickness was measured with a thickness gauge. Each value is presented as the mean ± SEM and analyzed with Tukey’s post hoc test from three independent experiments. ^###^ *p* < 0.001 vs. no treatment control group; ** *p* < 0.01 and *** *p* < 0.001 vs. anti-DNP IgE/BSA treatment only group.

To confirm the effect of the TAAR extract on the allergic response in vivo, a PCA-induced mouse model was used. This local cutaneous anaphylaxis model is a type I hypersensitive allergic reaction [26]. Systemic allergic reactions were induced using an anti-DNP IgE and a DNP-BSA-Evans blue mixture. The allergic response was quantified by measuring the amount of Evans blue pigment that diffused into the local tissue. Mice sensitized with anti-DNP IgE showed relatively high pigmentation compared with mice in the control group. However, the pigmentation of the ears was reduced in mice that were orally administered TAAR extract, TA extract, AR extract, and dexamethasone (Figure 9A and S1). In particular, it can be confirmed that the oral administration of the mixed extract of wheatgrass and aronia showed a better inhibitory effect than the oral administration of wheatgrass or aronia single extract at the same concentration. Then, ear thickness was measured for each mouse (Figure 9B), and dye was extracted from the ears of each mouse and quantified (Figure 9C). The results indicate that the TAAR extract significantly inhibits allergic reactions better than wheatgrass or aronia-alone extract.

## 3. Materials and Methods

### 3.1. Chemicals and Reagents

Standard samples (chlorogenic acid and γ-aminobutyric acid (GABA)) for high-performance liquid chromatography (HPLC), 3-(4,5-dimethylthiazol-2-yl)-2,5-diphenyltetrazolium bromide (MTT) solution, lipopolysaccharide (LPS), vitamin C, and dexamethasone were obtained from Sigma-Aldrich (St. Louis, Missouri, USA). Recombinant human interferon (IFN)-γ and tumor necrosis factor (TNF)-α were obtained from BioLegend (San Diego, CA, USA). Fetal bovine serum (FBS), Dulbecco’s modified Eagle medium (DMEM), and Roswell Park Memorial Institute (RPMI) 1640 medium were purchased from Gibco BRL (Rockville, MD, USA). Enzyme-linked immunosorbent assay (ELISA) kit for histamine was obtained from Abcam (Cambridge, UK) for histamine. Antibodies against p-p38, p38, p-ERK, ERK, p-JNK, JNK, p-Lyn, Lyn, p-Syk, Syk, p-Fyn, Fyn, p-PLCγ1, PLCγ1, p-PKCδ, PKCδ, NF-κB p65, Lamin B, Nrf2, HO-1, NQO1, iNOS, COX-2, and β-actin were purchased from Cell Signaling Technology (Danvers, MN, USA) or Santa Cruz (Dallas, TX, USA).

### 3.2. Preparation of Wheatgrass-and-Aronia-Mixed Extract

Aronia was imported from Poland, and wheatgrass seeds were provided by the National Institute of Crop Science and grown at the Saemangeum Biotechnology Center located in Gimje, Republic of Korea. We mixed wheatgrass and aronia in various ratios and extracted twice for 4 h at 60 °C using 30% ethanol, which was eight times the total amount. The extracts were first filtered, concentrated at 600–700 mm/Hg at 60–70 °C, and then sterilized for 60 min at 95 °C. The extracts were lyophilized after a secondary filtration. The prepared wheatgrass-and-aronia-mixed extracts were stored at −20 °C and used after dissolving in purified water for subsequent experiments.

### 3.3. HPLC Analysis

The conditions for the HPLC analysis of chlorogenic acid and GABA in wheatgrass and aronia (1:3) mixed (TAAR) extracts were shown as follows: HPLC analyse of Chlorogenic acid was conducted using an Agilent 1200 system equipped with UV detector. A UV detector at 327 nm and an analytical Capcell pack MG (4.6 mm × 150 mm, 5 μm) column were used for quantification. The temperature of the column was 30 °C, and the mobile phase flow rate was 0.7 mL/min. The eluent was a gradient of solvent (A) = 0.1% Phosphoric acid and solvent (B) = 100% MeOH. The run time was 55 min, and the gradient was as follows: (A)/(B) = 80/20 (0–55 min). HPLC analysis of γ-aminobutyric acid (GABA) was conducted using an Agilent LC system equipped with PDA. The PDA detector at 338 nm and 262 nm and an analytical Capcellpak UG120 C18 (250 mm × 4.6 mm, 5 μm) column was used for quantification. The temperature of column was 40 °C, and the mobile phase flow rate was 0.7 mL/min. The eluent was a gradient of solvent (A) = 40 mM NaH_2_PO_4_ (pH 7.8) and solvent (B) = ACN:MeOH:DW (45:45:10). The run time was 50 min, and the gradient was as follows: (A)/(B) = 95/5 (0–35 min) → (A)/(B) = 65/35 (35–45 min) → (A)/(B) = 44/56 (45–46 min) → (A)/(B) = 0/100 (46–50 min).

### 3.4. DPPH Radical Scavenging Activity

The DPPH radical-reducing power was measured in wheatgrass-and-aronia-mixed extracts. Five hundred microliters of various ratios of wheatgrass-and-aronia-mixed extracts (mixing ratios of wheatgrass/aronia = 1:1, 1:2, 1:3) or vitamin C (4 μg/mL, positive control) were added to 2 mL 0.15 mM DPPH solution. Each sample was thoroughly mixed by vortexing twice for 10 s and incubated for 40 min in the dark at room temperature. The absorbance was then measured at 517 nm using a microplate reader (Winooski, VT, USA) and calculated using the following equation:

DPPH radical scavenging activity (%) = {1 − (Sample absorbance/Control absorbance)} × 100

### 3.5. ABTS Radical Scavenging Assay

Solutions of 2.6 mM potassium persulfate and 7.4 mM ABTS were mixed in a 1:1 ratio, adjusted to pH 7.4, and allowed to react at room temperature for one day to prepare an ABTS radical scavenger. Aliquots of 20 μL wheatgrass-and-aronia-mixed extracts (wheatgrass/aronia = 1:1, 1:2, and 1:3) or vitamin C (positive control) were reacted with 180 μL of ABTS solution at room temperature for 10 min in a 96-well microplate before the absorbance was measured at 734 nm using a microplate reader (Winooski, VT, USA). ABTS radical scavenging activity was calculated using the following equation:

ABTS radical scavenging activity (%) = {1 − (Sample absorbance/Control absorbance)} × 100

### 3.6. Fluorescent Recovery after Photobleaching (FRAP) Assay

Sodium acetate and acetic acid were well mixed with stirring before the pH was adjusted to 3.6 to prepare a 300 mM sodium acetate buffer solution. A 10 mM 2,4,6-tripyridyl-s-triazine (TPTZ) solution was prepared by mixing 40 mM HCl and TPTZ. The sodium acetate buffer (pH 3.6), 10 mM TPTZ solution, and 20 mM FeCl_3_ 6H_2_O (ferric chloride hexahydrate) were mixed in a ratio of 10:1:1 to prepare a reaction solution and warmed to 37 °C before use. Then, 30 μL of wheatgrass-and-aronia-mixed extracts (wheatgrass:aronia = 1:1, 1:2, 1:3) or vitamin C (positive control) was mixed with 900 μL warmed FRAP solution and 90 μL distilled water and allowed to react for 10 min before the absorbance was measured at 593 nm using a microplate reader (Winooski, VT, USA).

### 3.7. Cell Culture

The RAW 264.7 (mouse macrophages, ATCC; TIB-71) and RBL-2H3 (rat basophilic leukemia cells, ATCC; CRL-2256) cell lines were obtained from the American Type Culture Collection (Manassas, VA, USA). The human keratinocyte (HaCaT) cell line was obtained from the Korean Cell Bank. The cell lines were maintained in DMEM or RPMI 1640 media supplemented with 10% FBS, 100 units/mL penicillin, and 100 µg/mL streptomycin (Welgene, Republic of Korea) and incubated in a 5% CO_2_ humidified incubator at 37 °C.

### 3.8. MTT Assay

The MTT assay was used to measure the effects of wheatgrass-and-aronia-mixed extracts on RAW 264.7, HaCaT, or RBL-2H3 cell viability. The cells (1 × 10^4^ cells/well) were seeded into a 96-well plate and incubated with various concentrations of wheatgrass-and-aronia-mixed extracts for 24 h at 37 °C. Then, the plate was incubated with 20 μL 5 mg/mL MTT reagent for a further 4 h at 37 °C in the dark. The supernatants were discarded, and 150 µL dimethyl sulfoxide (DMSO) was used to solubilize the formazan crystals. The optical absorbance was measured at 570 nm using a microplate reader (Winooski, VT, USA).

### 3.9. Quantitative Real-Time PCR (qRT-PCR)

The RAW 264.7 and HaCaT cells were pre-treated with 20 μg/mL wheatgrass-and-aronia-mixed extracts (1:1, 1:2, 1:3, 2:1, and 3:1) for 2 h before being treated with LPS or TNF-α/IFN-γ for 30 min. Anti-DNP IgE-sensitized RBL-2H3 cells were pretreated for 1 h with the 0, 20, 40, and 80 μg/mL TAAR extract and stimulated with 100 ng/mL DNP-BSA for 30 min. Total RNA from each sample was isolated using 1 mL TRIzol reagent and quantified with spectrophotometry. The cDNA was synthesized using 2 μg RNA and a PrimeSuperScript™ II cDNA synthesis kit (Takara Bio, Inc., San Jose, CA, USA). The mRNA expression levels were determined using Power SYBR Green PCR Master Mix and the AB StepOne system (Applied Biosystems, Santa Clara, CA, USA). Primer sequences are listed in Table 1.

### 3.10. β-hexosaminidase Release Assay

An indicator of the degranulation–hexosaminidase release assay was performed to measure the anti-allergic effect of the TAAR extract, as previously described [27]. Briefly, 5 × 10^5^ cells/mL RBL-2H3 cells were seeded into 48-well plates and sensitized with 100 ng/mL anti-DNP-IgE for 24 h. The IgE-sensitized RBL-2H3 cells were pre-incubated with 0, 20, 40, and 80 μg/mL TAAR extract for 1 h before 100 ng/mL DNP-BSA was added, and the cells were stimulated for an additional 4 h. The culture supernatant was collected, and the 50 µL supernatant was transferred to a 96-well plate and incubated with 50 µL substrate buffer (p-NAG in 0.1 M citrate buffer, pH 4.5) for 1.5 h at 37 °C. The reaction was terminated by adding 200 µL stop solution (0.1 M NaHCO_3_/Na_2_CO_3_, pH 10), and the optical absorbance was measured at 405 nm using a microplate reader.

### 3.11. Histamine Release

The histamine release was measured by collecting the supernatant after stimulation with DNP-BSA for 6 h under the same conditions as β-hexosaminidase release. Histamine release concentrations were measured using an ELISA kit (Abcam, Cambridge, UK) following the manufacturer’s instructions.

### 3.12. Western Blotting

The RBL-2H3 cells were harvested with PBS and centrifuged at 12,000× *g* for 15 min at 4 °C to remove the supernatant. Total protein was extracted using a cold pro-prep protein lysis solution containing protease and phosphatase inhibitors on ice. The extracted protein was quantified in equivalent amounts using Bradford reagent at 595 nm. Equal quantities of protein samples (30 μg) were loaded and separated by 10% sodium dodecyl sulfate (SDS)-sulfate-polyacrylamide gel electrophoresis (PAGE) for 2 h at 110 V and transferred to polyvinylidene fluoride (PVDF) membranes (Merck Millipore, Seoul, Republic of Korea). The membranes were blocked with 5% skim milk or 5% BSA in 1× Tris-buffered saline containing 0.1% Tween-20 (TBST) for 1 h and incubated with 1:1000 diluted primary antibodies (p-p38, p38, p-ERK, ERK, p-JNK, JNK, p-Lyn, Lyn, p-Fyn, Fyn, p-Syk, Syk, p-PLCγ1, PLCγ1, p-PKCδ, PKCδ, NFκB p65, Lamin B, Nrf2, HO-1, NQO1, iNOS, COX-2, or β-actin) at 4 °C overnight. After incubation, the membranes were washed three times with TBST and incubated with 1:5000 diluted horseradish peroxidase (HRP)-conjugated secondary antibodies for 2 h at room temperature. The membranes were washed three times with TBST and visualized using enhanced chemiluminescence (ECL) solution. Membrane images were obtained using a Davinch in vivo and western imaging system (Davinch-K, Seoul, Republic of Korea).

### 3.13. Animals

Five-week-old male BALB/c mice (20–22 g) were purchased from SAMTAKO Bio (Osan, Republic of Korea). During the experiment, tap water and a standard laboratory diet were provided to the mice ad libitum. All mice were housed in a controlled animal room at 21–22 °C, 50–60% humidity, and a 12–12 h light/dark cycle. The care and treatment of the mice were performed with the approval of the Animal Experiment Ethics Committee of Jeonbuk National University (Ethics Committee approval number: JBNU 2022-014).

### 3.14. Induction of IgE-Mediated Passive Cutaneous Anaphylaxis (PCA) Mouse Model

The IgE-mediated PCA mouse model was established as previously described [27]. The mice were randomly divided into five groups of six mice each. Anti-DNP-IgE (0.5 μg/ear site) was intradermally injected by 20 μL into the skin of ear of mice using an insulin syringe and sensitized for 48 h. Then, 40 or 80 mg/kg TAAR extract, 80 mg/kg TA extract, 80 mg/kg AR extract, or 10 mg/kg dexamethasone (positive control) were orally administered to each group. After 1 h, 200 μL of 10 mg/mL DNP-BSA dissolved in 4% Evans blue (1:1) was injected into the tail vein. After challenge for 2 h (time course test; 30 min, 1 h, and 2 h), all mice were anesthetized with CO_2_, and ear thickness was measured three times. Stained ear tissue was collected, immersed in 1 mL 1 M KOH solution, and dissolved for 2 d. The reaction was stopped by adding 4 mL acetone/phosphoric acid mixture (5:13) before the optical absorbance was measured at 620 nm using a microplate reader.

### 3.15. Statistical Analysis

All data are presented as means ± SEM (standard error of the mean) of three individual experiments. Statistical significance was evaluated by one-way analysis of variance (ANOVA) with Tukey’s post hoc analysis using GraphPad Prism (version 5.0; GraphPad Software, Inc., San Diego, CA, USA). *p* < 0.05 was considered a statistically significant difference.

## 4. Discussion

Mast cells are involved in various allergic reactions, including allergic rhinitis, inflammatory diseases, and asthma. These cells are located in many parts of the body exposed to the external environment, such as the respiratory system, digestive system, and skin [28]. When exposed to an external antigen, antigen-specific IgE is produced in the body, and activated mast cells express FcεRI, which combines with IgE. When newly exposed to an antigen, IgE-mediated activation occurs, and mast cells are degranulated, releasing histamine and secreting inflammatory cytokines that initiate an allergic response [29]. These IgE-dependent response mediators produced by extensive mast cell activation are risk factors for allergic disorders that can lead to potentially life-threatening anaphylactic reactions [30]. Currently, drugs commonly used to treat acute or chronic allergic reactions caused by mast cell activation are not completely fundamental and have many side effects; therefore, it is necessary to develop natural product-based treatments.

Based on studies on the pharmacological activities of various natural products, we selected wheatgrass and aronia as candidate natural products for the treatment of allergic diseases and focused on their potential anti-allergic effects of mixed extracts. After mixing and extracting wheatgrass and aronia at various mixing ratios, comparative analyses of their anti-inflammatory and antioxidant effects were conducted. Our analysis identified the 1:3 wheatgrass-and-aronia-mixed extract (TAAR) as the solution with the best effect. In addition, we were able to confirm that the mixed extract of wheatgrass and aronia had a synergistic effect compared to the single extract of wheatgrass or aronia (Supplementary Figure S2).

Phytochemical analysis confirmed the index components of the selected TAAR extract, which contained chlorogenic acid and GABA components. According to previous studies, both chlorogenic acid and GABA could alleviate mast cell activation and allergic inflammatory responses [31,32]; therefore, we hypothesized that TAAR extract could play a role in attenuating IgE-mediated mast cell activation and allergic inflammatory responses and could be used as a candidate for the treatment of allergic disorders in the future.

The RBL-2H3 cell line was used to study anti-allergic activity and related mechanisms. Therefore, after activating RBL-2H3 cells with anti-DNP IgE and DNP-BSA treatments, we examined the anti-allergic effects of the TAAR extract.

Mast cells have secretory granules filled with molecules such as histamine, β-hexosaminidase, and pro-inflammatory cytokines that are secreted as part of allergic reactions. The identification of mast cell degranulation using the secretion of β-hexosaminidase in RBL-2H3 cells is commonly used to confirm the effect on the anti-allergic response; histamine, which is secreted with β-hexosaminidase, leads to allergic inflammation and causes various allergic symptoms [24,33]. In addition, pro-inflammatory cytokines such as those secreted by mast cells are closely related to the initiation and progression of allergic disorders. TNF-α and IL-1β are potent inflammatory-related cytokine and the major cytokines in activated mast cells. IL-4 is known to induce B cell differentiation, IgE production, and anaphylaxis [34]. IL-6 plays an important role in the acute allergic inflammatory response, induces histamine production, and FcεRI expression in mast cells [35]. In the present study, TAAR extract inhibited the secretion of histamine and β-hexosaminidase and reduced the production of pro-inflammatory cytokines in RBL-2H3 cells activated with anti-DNP IgE and DNP-BSA. When FcεRI expressed on the surface of activated mast cells binds to IgE, it induces the MAPK signaling pathway and NF-εB activation [36]. The MAPKs (p38, ERK, and JNK) participate in a signaling pathway that regulates the activation, differentiation, and degranulation of immune cells, such as mast cells. The activation of p38 results in the production and secretion of IL-4, whereas the activation of ERK and JNK contributes to the production of pro-inflammatory cytokines, including IL-6 and TNF-α, in mast cells [37]. In addition, many previous studies have shown that NF-κB is an important transcription factor in allergic diseases, and its activation is essential for the production and secretion of cytokines in mast cells [38]. As activation of these pathways plays an important role in allergic pro-inflammatory mediators formation, we investigated the inhibitory effects of TAAR extract on these allergy-related mechanisms [24]. Our results confirmed that TAAR extract targeted and modulated the MAPK signaling pathway and NF-κB nuclear translocation. This suggested that the TAAR extract effectively alleviated inflammatory and allergic reactions by suppressing the expression of pro-inflammatory mediators such as iNOS and COX-2 through the inhibition of these regulatory pathways in activated mast cells.

Chronic diseases such as allergies are associated with a lack of antioxidants. When mast cells are activated by external stimuli, oxidative stress is induced [39]. The Nrf2 signaling pathway is a typical antioxidant-related mechanism, and HO-1 and NQO1, which are cell protective enzymes against oxidative stress, are regulated by Nrf2. We evaluated the potential of the TAAR extract as an antioxidant in mast cells under increased oxidative stress. In this study, it was confirmed that the TAAR extract could act as an antioxidant activating the antioxidant system in mast cells by up-regulating the Nrf2 signaling pathway and increasing the expression of HO-1 and NQO1.

Binding of IgE to FcεRI on mast cells induces the immediate activation of the Lyn/Fyn/Syk pathway and PI3K/PLCγ1/PKCδ signaling cascade. As previously reported, IgE-mediated degranulation is regulated by the Lyn/Fyn/Syk pathway, which is an FcεRI-mediated signaling cascade in mast cells [40]. In addition, the PLCγ1/PKCδ pathway contributes to mast cell degranulation, stimulating the secretion of granular substances such as histamine, β-hexosaminidase, and inflammatory cytokines [41]. In our study, we confirmed that TAAR extract modulates the activation of the FcεRI signaling cascades by suppressing the phosphorylation of Lyn, Fyn, Syk, PLCγ1, and PKCδ in activated mast cells. Thus, TAAR extract could be applied in the treatment of allergic diseases as an effective way to alleviate the allergic inflammatory response through this pathway.

The PCA model is a well-established animal model for examining type I hypersensitivity reactions [26]. Immediate hypersensitivity to allergen exposure in IgE-sensitized mice can affect one or more organ systems, resulting in the release of mediators from mast cells within minutes, leading to anaphylactic symptoms such as edema, vascular permeability, and erythema [42]. Therefore, when an allergen is injected intradermally into the ear of IgE-sensitized mice, an initial allergic reaction called immediate hypersensitivity occurs in PCA mouse models, which is marked by increased vascular permeability and edema in the mouse ear. Our data showed that TAAR extract improved edema and ear pigmentation caused by anaphylactic symptoms in a PCA mouse model. These results indicate that the TAAR extract effectively controlled mast cell activation in skin with type I hypersensitivity.

## 5. Conclusions

Among the different mixing ratios of wheatgrass and aronia extracts tested, a mixing ratio of 1:3 had the most significant anti-inflammation and antioxidant effects. Therefore, we investigated the antiallergic effects of the TAAR extract in mast cell lines and a mouse model. This study demonstrated that TAAR extract effectively inhibited IgE-mediated allergic responses in vitro and in vivo through the immediate activation of the Lyn/Fyn/Syk pathway and the PLCγ1/PKCδ intracellular calcium ion level regulating pathway. In addition, It was confirmed that the treatment of TAAR extract in activated mast cells inhibited the secretion of inflammatory mediators by down-regulating MAPK signaling and the NF-κB nuclear translocation pathway and increased the expression of antioxidant-related factors by activating the Nrf2 signaling pathway. Therefore, our results suggest that TAAR extract is a potential therapeutic candidate for treating allergic reactions.

## Figures and Tables

**Table 1:** Primer sequences for qRT-PCR.

Gene	Forward	Reverse
mTNF-α	TAGCCAGGAGGGAGAACAGA	TTTTCTGGAGGGAGATGTGG
mIL-1β	CTCCATGAGCTTTGTACAAGG	TGCTGATGTACCAGTTGGGG
hTNF-α	TTGGAGTGATCGGCCCCCAG	ACAGGCTTGTCACTCGGGGTT
hIL-1β	CAGCTCTCTCCTTTCAGGGCCA	GGCCGTGGTTTCTGTCAGGC
rTNF-α	GAAAGCATGATCCGAGATGTGG	TCATACCAGGGCTTGAGCTCA
rIL-1β	CCCTGCAGCTGGAGAGTGTGG	TGTGCTCTGCTTGAGTGCT
rIL-6	GGAGACTTCACAGAGGATAC	CCATTAGGAGAGCATTGGAAG
rIL-4	ACCCTGTTCTGCTTTCTC	GTTCTCCGTGGTGTTCCT
mGAPDH	CATGGCCTTCCGTGTTC	CCTGGTCCTCAGTGTAGC
hGAPDH	GAAGGTGAAGGTCGGAGT	GAAGATGGTGATGGGATTTC
rGAPDH	AACGGCACAGTCAAGGCTGA	ACGCCAGTAGACTCCACGACAT

## Data Availability

The data are contained within the article and Supplementary Materials.

## Supplementary Materials

The following supporting information can be downloaded at: https://www.mdpi.com/article/10.3390/ijms241511979/s1.
