# Supplementary material for: Wheatgrass-and-Aronia-Mixed Extract Suppresses Immunoglobulin E-Mediated Allergic Reactions In Vitro and In Vivo
Source: Int J Mol Sci. 2023 Jul 26;24(15):11979. doi: 10.3390/ijms241511979 (PMC10419027; doi:10.3390/ijms241511979)
Supplement: Supplementary file 1 [file ijms-24-11979-s001.zip › ijms-2487910-supplementary.pdf]

Supplementary Figure S1.

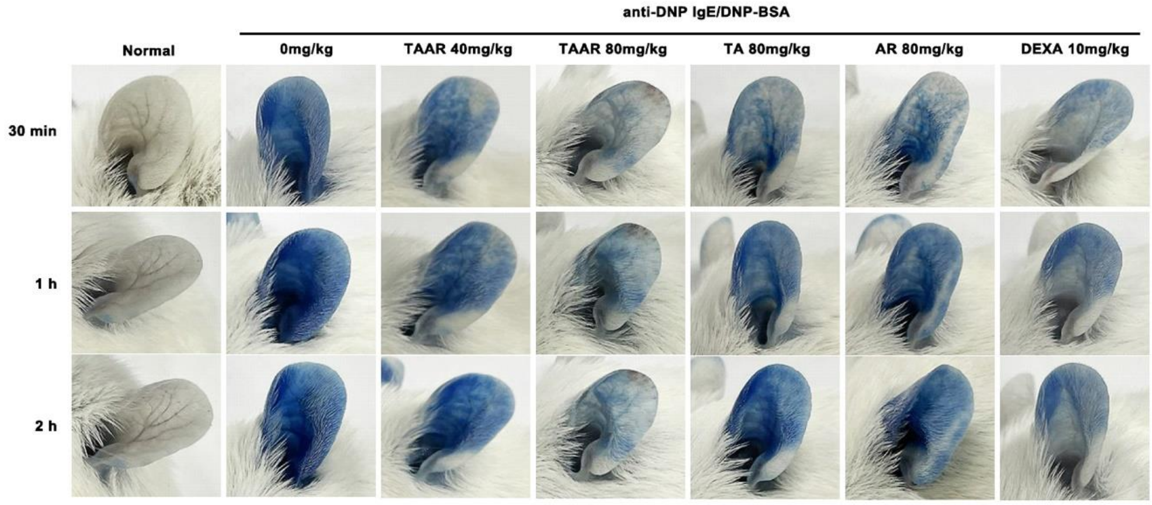

Supplementary Figure S1. Effects of TAAR, wheatgrass, and aronia extracts on IgE-mediated passive cutaneous anaphylaxis mouse model. Representative pictures of mouse ear according to the time course (10 min, 30 min, 1 h, and 2 h) in each group.

Supplementary Figure S2.

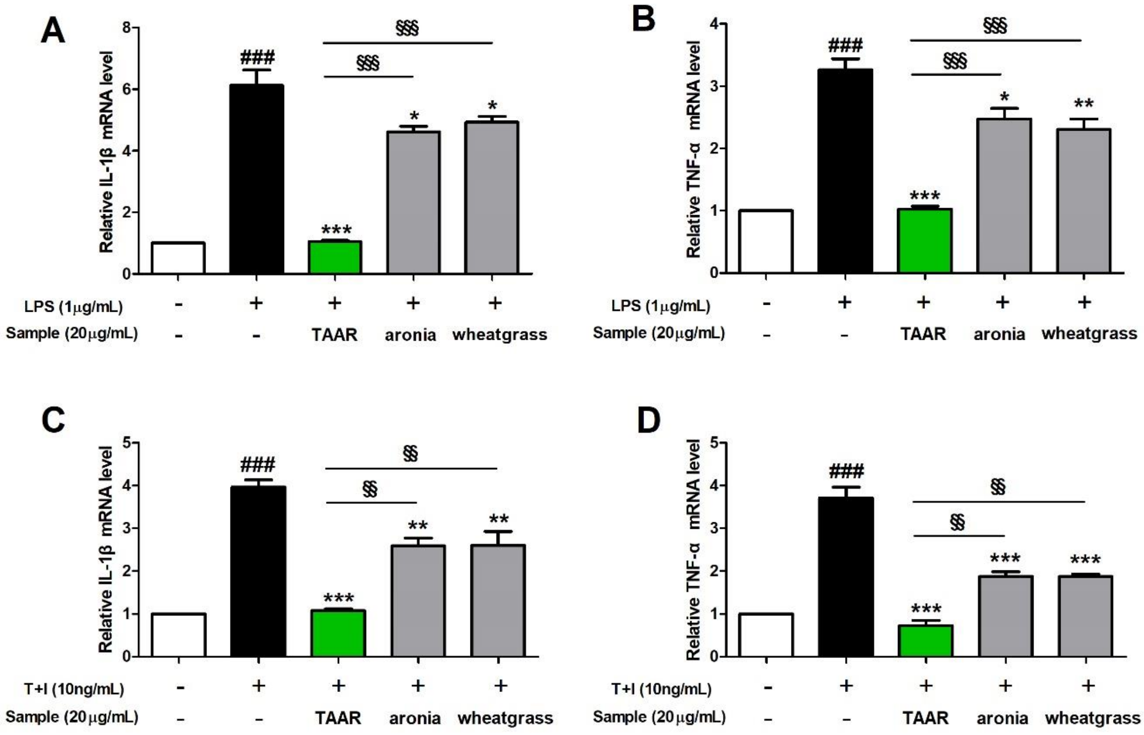

Supplementary Figure S2. Comparison of anti-inflammatory effects of wheatgrass and aronia mixed extract (TAAR) and wheatgrass or aronia alone extract. (A and B) The mRNA expression levels of pro-inflammatory cytokines in response to treatment with TAAR and wheatgrass or aronia alone extract in LPS-induced RAW 264.7 cells. (C and D) The mRNA expression levels of pro-inflammatory cytokines in response to treatment with TAAR and wheatgrass or aronia alone extract in TNF-α/IFN-γ-induced HaCaT cells. Values are represented as the mean ± SEM and analyzed by Tukey's *post hoc* test from three independent experiments. $^{###}p < 0.001$ vs. no treatment control group; $^{*}p < 0.05$, $^{**}p < 0.01$, and $^{***}p < 0.001$ vs. LPS or TNF-α/IFN-γ treatment only group. $^{§§}p < 0.01$, and $^{§§§}p < 0.001$ vs. TAAR treatment group.
